# Crystal structure of bis­{2-[(2-hy­droxy­eth­yl)amino]­ethanol-κ^3^ *O*,*N*,*O*′}copper(II) terephthalate

**DOI:** 10.1107/S1600536814022272

**Published:** 2014-10-18

**Authors:** Ya-Ping Li, Dajun Sun, Julia Ming, Liying Han, Guan-Fang Su

**Affiliations:** a Department of Ophthalmology, The Second Hospital of Jilin University, 218 Ziqiang Street, Changchun 130041, People’s Republic of China; b Department of Vascular Surgery, The China-Japan Union Hospital of Jilin University, Changchun 130033, People’s Republic of China; c St Erik’s Eye Hospital, Karolinska Institutet, Polhemsgatan 50, SE-112 82 Stockholm, Sweden; d Department of Gynecology, The Second Hospital of Jilin University, 218 Ziqiang Street, Changchun 130041, People’s Republic of China

**Keywords:** crystal structure, copper(II) chelate complex, terephthalate

## Abstract

The mol­ecular components of the title salt, [Cu(C_4_H_11_NO_2_)_2_](C_8_H_4_O_4_), are one Cu^II^ cation *O*,*N*,*O*′-chelated by two tridentate 2-[(2-hy­droxy­eth­yl)amino]­ethanol ligands, and a terephthalate counter-dianion, located about a centre of inversion. The complex Cu^II^ cation is located about a centre of inversion and shows typical Jahn–Teller distortion, with two short Cu—O and two short Cu—N bonds in the equatorial plane and two long Cu—O bonds to the axial atoms. The cations are arranged in sheets parallel to (100), with the centrosymmetric terephthalate anions located between the sheets. Each anion is the acceptor of four O—H⋯O and two N—H⋯O hydrogen bonds, forming a three-dimensional network structure.

## Related literature   

For related copper(II) compounds with terephthalate anions, see: Abbaszadeh *et al.* (2012 ▶); Al-Hashemi *et al.* (2010 ▶).
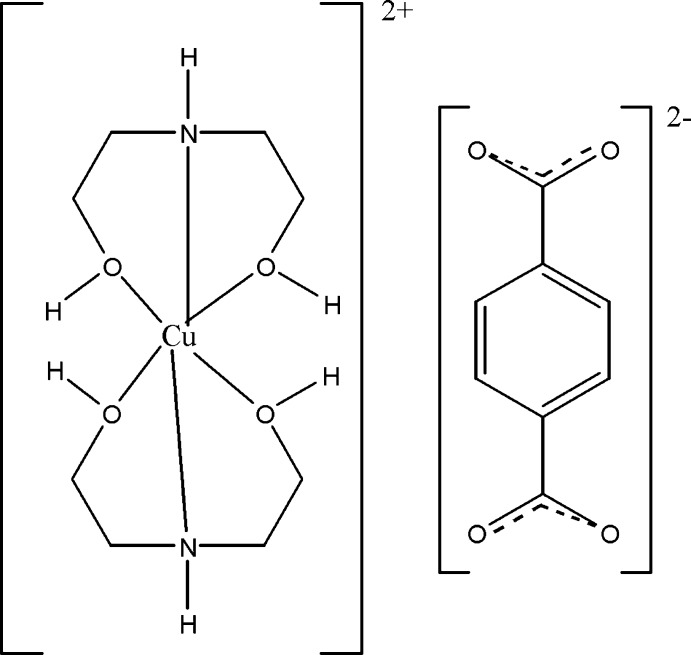


## Experimental   

### Crystal data  

[Cu(C_4_H_11_NO_2_)_2_](C_8_H_4_O_4_)
*M* *_r_* = 437.93
Monoclinic, 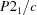

*a* = 8.6013 (9) Å
*b* = 9.0398 (9) Å
*c* = 11.5732 (12) Å
β = 91.695 (2)°
*V* = 899.47 (16) Å^3^
*Z* = 2
Mo *K*α radiation
μ = 1.26 mm^−1^
*T* = 293 K
0.29 × 0.27 × 0.26 mm

### Data collection  

Bruker SMART APEXII CCD diffractometer
Absorption correction: multi-scan (*SADABS*; Bruker, 2002 ▶) *T*_min_ = 0.728, *T*_max_ = 0.812
4784 measured reflections
1780 independent reflections
1611 reflections with *I* > 2σ(*I*)
*R*_int_ = 0.034

### Refinement  

*R*[*F* ^2^ > 2σ(*F* ^2^)] = 0.030
*wR*(*F* ^2^) = 0.080
*S* = 1.08
1780 reflections
133 parameters
3 restraints
H atoms treated by a mixture of independent and constrained refinement
Δρ_max_ = 0.31 e Å^−3^
Δρ_min_ = −0.83 e Å^−3^

### 

Data collection: *APEX2* (Bruker, 2002 ▶); cell refinement: *SAINT* (Bruker, 2002 ▶); data reduction: *SAINT*; program(s) used to solve structure: *SHELXS97* (Sheldrick, 2008 ▶); program(s) used to refine structure: *SHELXL97* (Sheldrick, 2008 ▶); molecular graphics: *SHELXL97*; software used to prepare material for publication: *SHELXTL* (Sheldrick, 2008 ▶) and *publCIF* (Westrip, 2010 ▶).

## Supplementary Material

Crystal structure: contains datablock(s) global, I. DOI: 10.1107/S1600536814022272/wm5069sup1.cif

Structure factors: contains datablock(s) I. DOI: 10.1107/S1600536814022272/wm5069Isup2.hkl

**Fig. 1.**
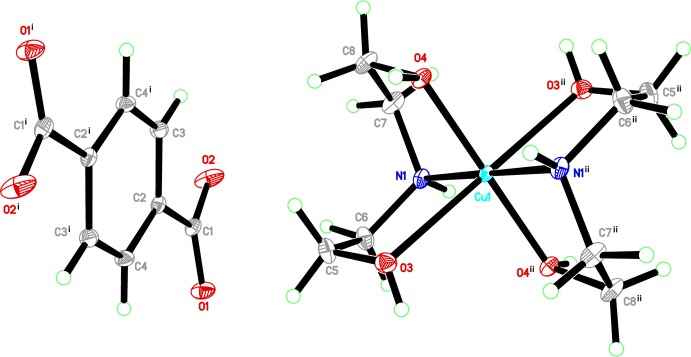
The molecular components of the title compound. Displacement ellipsoids are drawn at the 30% probability level. [Symmetry codes: (i) 3-x, 1-y, 2-z; (ii) 2-x, -y, 2-z.]

. DOI: 10.1107/S1600536814022272/wm5069fig1.tif
The mol­ecular components of the title compound. Displacement ellipsoids are drawn at the 30% probability level. [Symmetry codes: (i) 3-x, 1-y, 2-z; (ii) 2-x, −y, 2-z.]

**Fig. 2.**
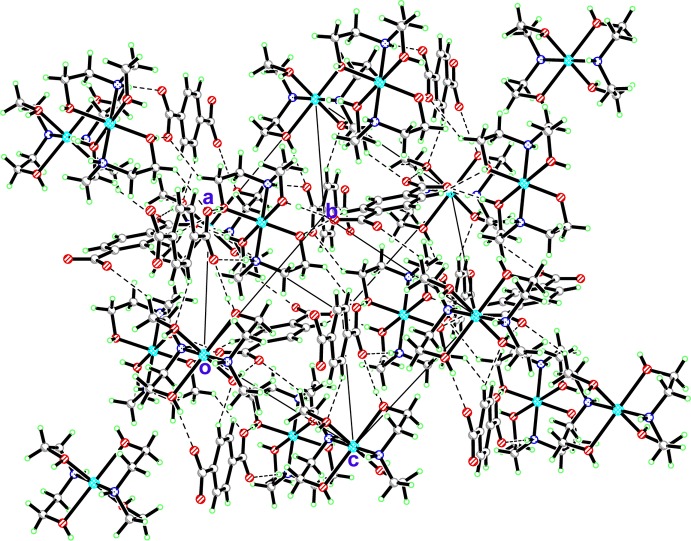
The packing of the molecular components in the title compound. N—H···O and O—H···O hydrogen bonds are shown by dashed lines.

. DOI: 10.1107/S1600536814022272/wm5069fig2.tif
The packing of the mol­ecular components in the title compound. N—H⋯O and O—H⋯O hydrogen bonds are shown by dashed lines.

CCDC reference: 1028231

Additional supporting information:  crystallographic information; 3D view; checkCIF report

## Figures and Tables

**Table 1:** Hydrogen-bond geometry (, )

*D*H*A*	*D*H	H*A*	*D* *A*	*D*H*A*
N1H1*A*O1^i^	0.85(2)	2.08(2)	2.9196(19)	168(2)
O4H4*A*O2^ii^	0.84(2)	1.66(2)	2.496(2)	180(3)
O3H3*A*O1^iii^	0.81(2)	1.88(2)	2.6803(19)	167(2)

Symmetry codes: (i) 
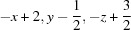
; (ii) 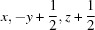; (iii) 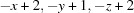.

## supplementary crystallographic information

### S1. Preparation

The synthesis was performed under hydro­thermal conditions. A mixture of Cu(CH_3_COO)_2_·2H_2_O, (0.2 mmol, 0.046 g), 2-(2-hy­droxy-ethyl­amine) (0.4 mmol, 0.043 g), sodium terephthalate (0.2 mmol, 0.042 g) and water (20 ml) in a 30 ml stainless steel reactor with a Teflon liner were heated from 293 to 433 K in 2 h, and a constant temperature was maintained at 433 K for 72 h, after which the mixture was cooled to 298 K. Blue crystals of the title compound were recovered from the reaction.

### S2. Refinement

All C—H H atoms were positioned with idealized geometry and refined with *U*_iso_(H) = 1.2*U*_eq_(C) using a riding model. The hy­droxy H-atoms and amine H atoms were located in a difference Fourier map and were refined with O—H or N—H distances restrained to 0.85 (3) Å and with *U*_iso_(H) = 1.5*U*_eq_(N,O).

### Crystal data

**Table:**

[Cu(C_4_H_11_NO_2_)_2_](C_8_H_4_O_4_)	*F*(000) = 458
*M**_r_* = 437.93	*D*_x_ = 1.617 Mg m^−^^3^
Monoclinic, *P*2_1_/*c*	Mo *K*α radiation, λ = 0.71073 Å
Hall symbol: -P 2ybc	Cell parameters from 1780 reflections
*a* = 8.6013 (9) Å	θ = 1.7–22.8°
*b* = 9.0398 (9) Å	µ = 1.26 mm^−^^1^
*c* = 11.5732 (12) Å	*T* = 293 K
β = 91.695 (2)°	Block, blue
*V* = 899.47 (16) Å^3^	0.29 × 0.27 × 0.26 mm
*Z* = 2	

### Data collection

**Table:**

Bruker SMART APEXII CCD diffractometer	1780 independent reflections
Radiation source: fine-focus sealed tube	1611 reflections with *I* > 2σ(*I*)
Graphite monochromator	*R*_int_ = 0.034
phi and ω scans	θ_max_ = 26.1°, θ_min_ = 2.4°
Absorption correction: multi-scan (*SADABS*; Bruker, 2002)	*h* = −10→8
*T*_min_ = 0.728, *T*_max_ = 0.812	*k* = −11→11
4784 measured reflections	*l* = −12→14

### Refinement

**Table:**

Refinement on *F*^2^	Primary atom site location: structure-invariant direct methods
Least-squares matrix: full	Secondary atom site location: difference Fourier map
*R*[*F*^2^ > 2σ(*F*^2^)] = 0.030	Hydrogen site location: inferred from neighbouring sites
*wR*(*F*^2^) = 0.080	H atoms treated by a mixture of independent and constrained refinement
*S* = 1.08	*w* = 1/[σ^2^(*F*_o_^2^) + (0.0446*P*)^2^ + 0.3198*P*] where *P* = (*F*_o_^2^ + 2*F*_c_^2^)/3
1780 reflections	(Δ/σ)_max_ < 0.001
133 parameters	Δρ_max_ = 0.31 e Å^−^^3^
3 restraints	Δρ_min_ = −0.83 e Å^−^^3^

### Special details

**Table:**

Geometry. All esds (except the esd in the dihedral angle between two l.s. planes) are estimated using the full covariance matrix. The cell esds are taken into account individually in the estimation of esds in distances, angles and torsion angles; correlations between esds in cell parameters are only used when they are defined by crystal symmetry. An approximate (isotropic) treatment of cell esds is used for estimating esds involving l.s. planes.
Refinement. Refinement of F^2^ against ALL reflections. The weighted R-factor wR and goodness of fit S are based on F^2^, conventional R-factors R are based on F, with F set to zero for negative F^2^. The threshold expression of F^2^ > 2sigma(F^2^) is used only for calculating R-factors(gt) etc. and is not relevant to the choice of reflections for refinement. R-factors based on F^2^ are statistically about twice as large as those based on F, and R- factors based on ALL data will be even larger.

### Fractional atomic coordinates and isotropic or equivalent isotropic displacement parameters (Å^2^)

**Table:**

	*x*	*y*	*z*	*U*_iso_*/*U*_eq_	
C1	1.3193 (2)	0.5638 (2)	0.79022 (15)	0.0172 (4)	
C2	1.4142 (2)	0.5319 (2)	0.89901 (16)	0.0152 (4)	
C3	1.5213 (2)	0.4163 (2)	0.90128 (16)	0.0182 (4)	
H3	1.5359	0.3603	0.8351	0.022*	
C4	1.3941 (2)	0.6154 (2)	0.99805 (15)	0.0181 (4)	
H4	1.3232	0.6932	0.9968	0.022*	
C5	1.1185 (2)	0.3121 (2)	1.02670 (17)	0.0256 (4)	
H5A	1.2286	0.2892	1.0319	0.031*	
H5B	1.1057	0.4155	1.0468	0.031*	
C6	1.0583 (2)	0.2870 (2)	0.90354 (17)	0.0235 (4)	
H6A	0.9534	0.3259	0.8956	0.028*	
H6B	1.1229	0.3417	0.8511	0.028*	
C7	1.2043 (2)	0.0697 (2)	0.82537 (15)	0.0233 (4)	
H7A	1.2537	0.1440	0.7784	0.028*	
H7B	1.1821	−0.0158	0.7769	0.028*	
C8	1.3139 (2)	0.0254 (2)	0.92433 (18)	0.0229 (4)	
H8A	1.3931	−0.0412	0.8968	0.027*	
H8B	1.3649	0.1123	0.9568	0.027*	
N1	1.05698 (17)	0.12937 (17)	0.86951 (12)	0.0159 (3)	
H1A	0.985 (2)	0.123 (2)	0.8179 (16)	0.024*	
O1	1.20598 (16)	0.64876 (16)	0.79517 (11)	0.0258 (3)	
O2	1.3607 (2)	0.49850 (17)	0.70051 (13)	0.0334 (4)	
O3	1.03789 (17)	0.22244 (15)	1.10678 (11)	0.0211 (3)	
H3A	0.956 (2)	0.259 (3)	1.127 (2)	0.032*	
O4	1.22477 (15)	−0.04666 (15)	1.01071 (11)	0.0163 (3)	
H4A	1.270 (3)	−0.031 (3)	1.0742 (16)	0.025*	
Cu1	1.0000	0.0000	1.0000	0.01196 (13)	

### Atomic displacement parameters (Å^2^)

**Table:**

	*U*^11^	*U*^22^	*U*^33^	*U*^12^	*U*^13^	*U*^23^
C1	0.0139 (9)	0.0228 (10)	0.0149 (9)	−0.0016 (8)	−0.0017 (7)	0.0012 (7)
C2	0.0130 (9)	0.0170 (8)	0.0156 (8)	−0.0019 (8)	−0.0020 (7)	0.0018 (7)
C3	0.0173 (9)	0.0203 (9)	0.0168 (8)	0.0013 (7)	−0.0014 (7)	−0.0034 (7)
C4	0.0162 (9)	0.0189 (9)	0.0192 (9)	0.0040 (8)	−0.0023 (7)	−0.0008 (7)
C5	0.0279 (11)	0.0218 (10)	0.0268 (10)	−0.0081 (8)	−0.0029 (8)	−0.0039 (8)
C6	0.0283 (11)	0.0185 (9)	0.0235 (10)	−0.0043 (8)	−0.0035 (8)	0.0038 (8)
C7	0.0184 (10)	0.0378 (12)	0.0137 (8)	0.0013 (9)	0.0006 (7)	0.0045 (8)
C8	0.0140 (10)	0.0371 (11)	0.0176 (9)	0.0004 (8)	0.0027 (8)	0.0047 (8)
N1	0.0135 (8)	0.0210 (8)	0.0128 (7)	−0.0019 (6)	−0.0052 (6)	0.0016 (6)
O1	0.0223 (8)	0.0351 (8)	0.0195 (7)	0.0124 (6)	−0.0071 (5)	−0.0038 (6)
O2	0.0252 (9)	0.0597 (13)	0.0149 (7)	0.0192 (7)	−0.0050 (6)	−0.0049 (6)
O3	0.0216 (7)	0.0223 (7)	0.0194 (6)	0.0015 (6)	−0.0005 (5)	−0.0037 (5)
O4	0.0126 (6)	0.0238 (7)	0.0125 (6)	0.0002 (6)	−0.0023 (5)	0.0010 (5)
Cu1	0.01017 (19)	0.0143 (2)	0.01133 (18)	−0.00020 (10)	−0.00122 (12)	0.00137 (10)

### Geometric parameters (Å, º)

**Table:**

C1—O1	1.243 (2)	C7—N1	1.482 (2)
C1—O2	1.255 (2)	C7—C8	1.515 (3)
C1—C2	1.508 (2)	C7—H7A	0.9700
C2—C4	1.387 (3)	C7—H7B	0.9700
C2—C3	1.392 (3)	C8—O4	1.434 (2)
C3—C4^i^	1.386 (2)	C8—H8A	0.9700
C3—H3	0.9300	C8—H8B	0.9700
C4—C3^i^	1.386 (2)	N1—Cu1	1.9830 (15)
C4—H4	0.9300	N1—H1A	0.847 (16)
C5—O3	1.427 (2)	O3—Cu1	2.3776 (13)
C5—C6	1.519 (3)	O3—H3A	0.814 (16)
C5—H5A	0.9700	O4—Cu1	1.9791 (13)
C5—H5B	0.9700	O4—H4A	0.835 (17)
C6—N1	1.478 (2)	Cu1—O4^ii^	1.9791 (13)
C6—H6A	0.9700	Cu1—N1^ii^	1.9830 (15)
C6—H6B	0.9700	Cu1—O3^ii^	2.3776 (13)
			
O1—C1—O2	124.81 (17)	C7—C8—H8A	110.0
O1—C1—C2	119.04 (16)	O4—C8—H8B	110.0
O2—C1—C2	116.13 (16)	C7—C8—H8B	110.0
C4—C2—C3	119.43 (17)	H8A—C8—H8B	108.4
C4—C2—C1	120.51 (17)	C6—N1—C7	116.30 (16)
C3—C2—C1	120.05 (17)	C6—N1—Cu1	111.45 (11)
C4^i^—C3—C2	120.13 (17)	C7—N1—Cu1	106.31 (11)
C4^i^—C3—H3	119.9	C6—N1—H1A	104.5 (15)
C2—C3—H3	119.9	C7—N1—H1A	110.2 (15)
C3^i^—C4—C2	120.43 (17)	Cu1—N1—H1A	107.9 (15)
C3^i^—C4—H4	119.8	C5—O3—Cu1	101.82 (10)
C2—C4—H4	119.8	C5—O3—H3A	113.5 (18)
O3—C5—C6	111.45 (16)	Cu1—O3—H3A	112.5 (18)
O3—C5—H5A	109.3	C8—O4—Cu1	113.63 (11)
C6—C5—H5A	109.3	C8—O4—H4A	106.7 (18)
O3—C5—H5B	109.3	Cu1—O4—H4A	116.8 (18)
C6—C5—H5B	109.3	O4—Cu1—O4^ii^	180.0
H5A—C5—H5B	108.0	O4—Cu1—N1^ii^	95.14 (6)
N1—C6—C5	113.19 (15)	O4^ii^—Cu1—N1^ii^	84.86 (6)
N1—C6—H6A	108.9	O4—Cu1—N1	84.86 (6)
C5—C6—H6A	108.9	O4^ii^—Cu1—N1	95.14 (6)
N1—C6—H6B	108.9	N1^ii^—Cu1—N1	180.0
C5—C6—H6B	108.9	O4—Cu1—O3^ii^	88.34 (5)
H6A—C6—H6B	107.8	O4^ii^—Cu1—O3^ii^	91.66 (5)
N1—C7—C8	110.78 (15)	N1^ii^—Cu1—O3^ii^	82.18 (5)
N1—C7—H7A	109.5	N1—Cu1—O3^ii^	97.82 (5)
C8—C7—H7A	109.5	O4—Cu1—O3	91.66 (5)
N1—C7—H7B	109.5	O4^ii^—Cu1—O3	88.34 (5)
C8—C7—H7B	109.5	N1^ii^—Cu1—O3	97.82 (5)
H7A—C7—H7B	108.1	N1—Cu1—O3	82.18 (5)
O4—C8—C7	108.27 (16)	O3^ii^—Cu1—O3	180.0
O4—C8—H8A	110.0		
			
O1—C1—C2—C4	−11.6 (3)	C8—O4—Cu1—N1	−1.66 (13)
O2—C1—C2—C4	169.71 (18)	C8—O4—Cu1—O3^ii^	96.35 (13)
O1—C1—C2—C3	167.30 (18)	C8—O4—Cu1—O3	−83.65 (13)
O2—C1—C2—C3	−11.4 (3)	C6—N1—Cu1—O4	−103.85 (13)
C4—C2—C3—C4^i^	0.4 (3)	C7—N1—Cu1—O4	23.81 (11)
C1—C2—C3—C4^i^	−178.47 (17)	C6—N1—Cu1—O4^ii^	76.15 (13)
C3—C2—C4—C3^i^	−0.4 (3)	C7—N1—Cu1—O4^ii^	−156.19 (11)
C1—C2—C4—C3^i^	178.46 (17)	C6—N1—Cu1—N1^ii^	5 (100)
O3—C5—C6—N1	−52.7 (2)	C7—N1—Cu1—N1^ii^	133 (100)
N1—C7—C8—O4	41.4 (2)	C6—N1—Cu1—O3^ii^	168.55 (12)
C5—C6—N1—C7	−85.3 (2)	C7—N1—Cu1—O3^ii^	−63.79 (12)
C5—C6—N1—Cu1	36.8 (2)	C6—N1—Cu1—O3	−11.45 (12)
C8—C7—N1—C6	82.9 (2)	C7—N1—Cu1—O3	116.21 (12)
C8—C7—N1—Cu1	−41.82 (18)	C5—O3—Cu1—O4	69.87 (12)
C6—C5—O3—Cu1	37.08 (18)	C5—O3—Cu1—O4^ii^	−110.13 (12)
C7—C8—O4—Cu1	−20.7 (2)	C5—O3—Cu1—N1^ii^	165.29 (12)
C8—O4—Cu1—O4^ii^	−53 (21)	C5—O3—Cu1—N1	−14.71 (12)
C8—O4—Cu1—N1^ii^	178.34 (13)	C5—O3—Cu1—O3^ii^	−40.8 (3)

Symmetry codes: (i) −*x*+3, −*y*+1, −*z*+2; (ii) −*x*+2, −*y*, −*z*+2.

### Hydrogen-bond geometry (Å, º)

**Table:**

*D*—H···*A*	*D*—H	H···*A*	*D*···*A*	*D*—H···*A*
N1—H1*A*···O1^iii^	0.85 (2)	2.08 (2)	2.9196 (19)	168 (2)
O4—H4*A*···O2^iv^	0.84 (2)	1.66 (2)	2.496 (2)	180 (3)
O3—H3*A*···O1^v^	0.81 (2)	1.88 (2)	2.6803 (19)	167 (2)

Symmetry codes: (iii) −*x*+2, *y*−1/2, −*z*+3/2; (iv) *x*, −*y*+1/2, *z*+1/2; (v) −*x*+2, −*y*+1, −*z*+2.
