# Supplementary figures and images for: Crystal structure of bis­{2-[(2-hy­droxy­eth­yl)amino]­ethanol-κ3 O,N,O′}copper(II) terephthalate
Source: Acta Crystallogr Sect E Struct Rep Online. 2014 Oct 18;70(Pt 11):m372–3. doi: 10.1107/S1600536814022272 (PMC4257266; doi:10.1107/S1600536814022272)

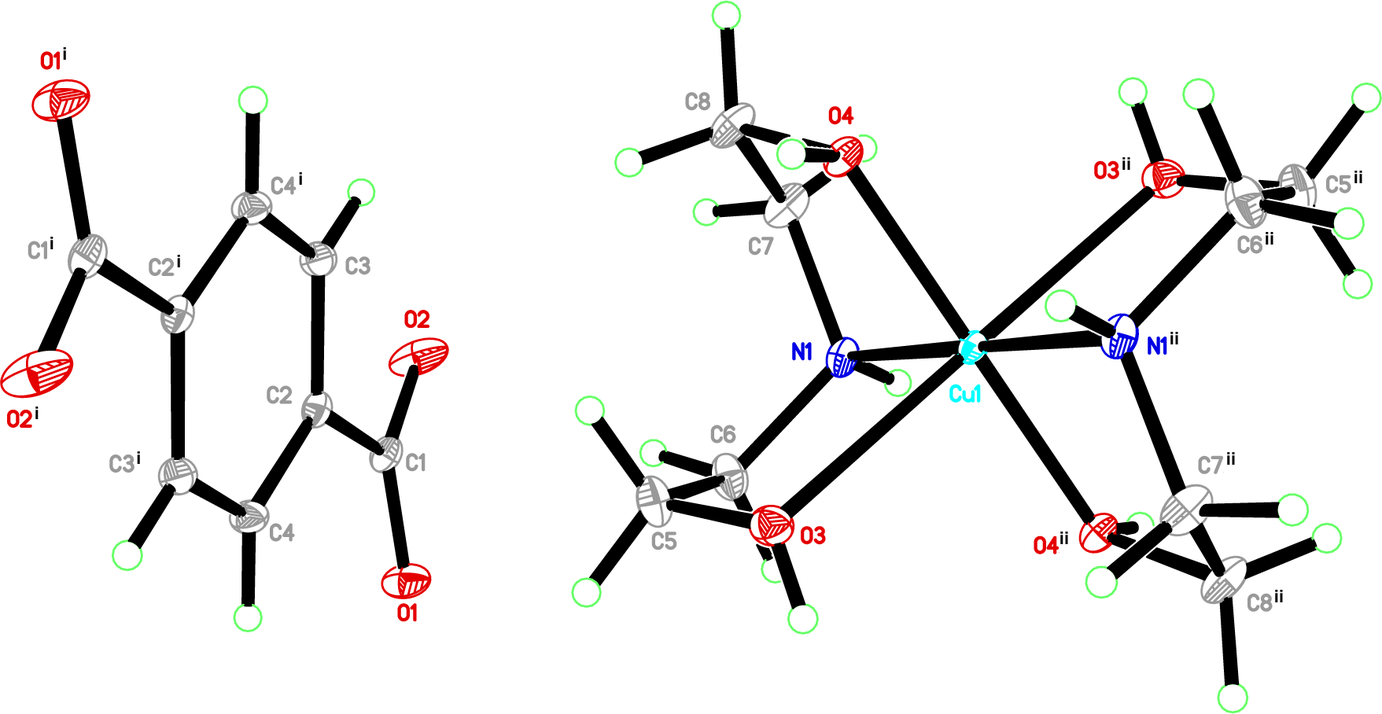

Supplement: Supplementary file 3 [file e-70-0m372-fig1.tif]

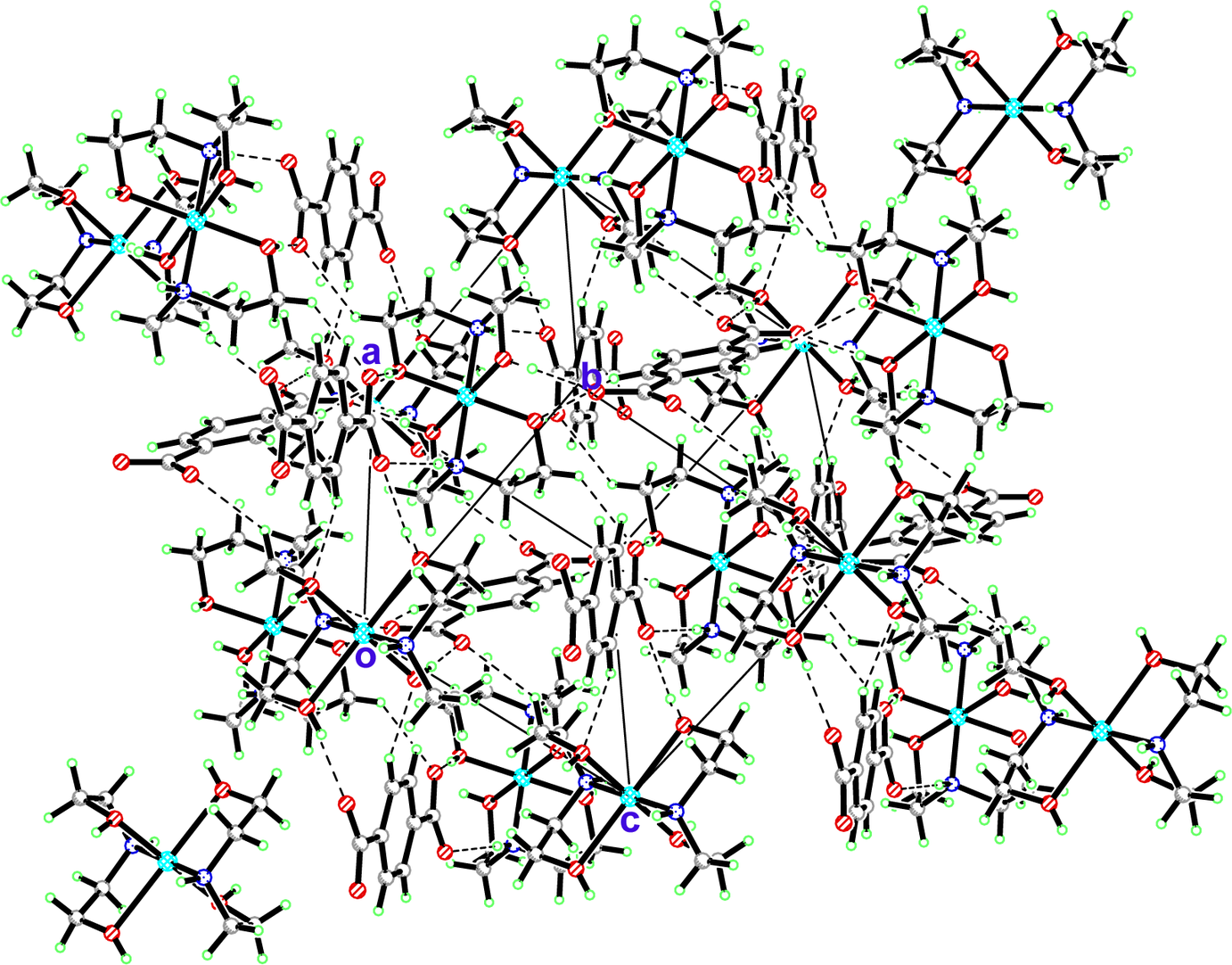

Supplement: Supplementary file 4 [file e-70-0m372-fig2.tif]
